# Longitudinal assessment of quality of life, neurocognition, and psychopathology in patients with low-grade glioma on first-line temozolomide: A feasibility study

**DOI:** 10.1093/noajnl/vdae084

**Published:** 2024-06-04

**Authors:** Amélie Darlix, Maëva Monnier, Florence Castan, Louise Coutant, Michel Fabbro, Ève Denis-Chammas, Mathilde Carrière, Nicolas Menjot-de-Champfleur, Valérie Rigau, Hugues Duffau, Estelle Guerdoux

**Affiliations:** Institut de Génomique Fonctionnelle, University of Montpellier, CNRS, INSERM, Montpellier, France; Department of Medical Oncology, Montpellier Cancer Institute (ICM), University of Montpellier, Montpellier, France; Biometrics Unit, Montpellier Cancer Institute (ICM), University of Montpellier, Montpellier, France; Biometrics Unit, Montpellier Cancer Institute (ICM), University of Montpellier, Montpellier, France; Department of Supportive Care, Psycho-Oncology Unit, Montpellier Cancer Institute (ICM), University of Montpellier, Montpellier, France; Department of Medical Oncology, Montpellier Cancer Institute (ICM), University of Montpellier, Montpellier, France; Department of Neuroradiology, University of Montpellier, CHU Montpellier, Montpellier, France; Department of Neuroradiology, University of Montpellier, CHU Montpellier, Montpellier, France; Department of Neuroradiology, University of Montpellier, CHU Montpellier, Montpellier, France; Institut de Génomique Fonctionnelle, University of Montpellier, CNRS, INSERM, Montpellier, France; Department of Pathology, University of Montpellier, CHU Montpellier, Montpellier, France; Institut de Génomique Fonctionnelle, University of Montpellier, CNRS, INSERM, Montpellier, France; Department of Neurosurgery, University of Montpellier, CHU Montpellier, Montpellier, France; Department of Supportive Care, Psycho-Oncology Unit, Montpellier Cancer Institute (ICM), University of Montpellier, Montpellier, France; Desbrest Institute of Epidemiology and Public Health (IDESP), INSERM, Montpellier Cancer Institute (ICM), University of Montpellier, France

**Keywords:** low-grade glioma, neurocognition, psychopathology, quality of life, temozolomide

## Abstract

**Background:**

The treatment timing and choice after neurosurgical resection in patients with newly diagnosed diffuse low-grade glioma (DLGG) remain controversial. Indeed, the effect of such treatments must be balanced with the possible side effects. This study evaluated the feasibility of longitudinal exhaustive quality of life (QoL) and neuropsychological assessments in patients with DLGG receiving first-line temozolomide.

**Methods:**

QoL, neurocognition, and psychological disorders were assessed prospectively until disease progression, using testing, clinician-reported, and self-reported questionnaires. The primary endpoint was the participation and adherence to this complete assessment at *Baseline* (before temozolomide initiation), months 6 and 12 of treatment, and month 6 post-treatment. The QoL and neuropsychological changes over time also were described.

**Results:**

Twenty-six of the twenty-nine eligible patients were enrolled (participation rate: 89.7%, 95% CI: 72.6–97.8). The adherence rate was 95.7% (95% CI: 78.1–99.9; *n* = 23 because 3 patients progressed in the first 12 months of treatment). Up to month 6 post-treatment, QoL and fatigue remained stable (EORTC QLQC30 and BN20, MFI-20); some specific symptoms were transitory. Both subjective (FACT-Cog) and objective (Z-scores of neurocognitive tests) neurocognitive outcomes remained stable or tended to improve. The percentage of patients with severe depression (BDI-II), anxiety (STAI-Y), or anger (STAXI-II) was stable over time.

**Conclusions:**

This prospective study demonstrated the feasibility of an exhaustive and longitudinal evaluation of QoL, neurocognition, and psychological disorders, with high acceptability by patients with DLGG undergoing chemotherapy. First-line temozolomide seems to have limited short-term effects on QoL and neurocognition. These findings must be confirmed in the long term and in a larger cohort.

## Lay Summary

Diffuse low-grade glioma (DLGG) is a common brain tumor. Some patients, but not all, receive a chemotherapy called temozolomide (TMZ) after surgery, but the effect of this treatment on quality of life is not clear. The authors of this study wanted to find out if a complete, longitudinal assessment of quality of life and neurocognition was feasible, and to explore how TMZ impacts these aspects in patients with DLGG. To do this, they assessed a group of 26 patients with LGG receiving TMZ using testing, clinician-reported and self-reported questionnaires before treatment and then again 6 and 12 months after starting treatment. Their results show these evaluation are feasible and TMZ seems to have limited short-term impact on quality of life and neurocognition, but larger studies and long-term evaluations are needed to confirm this finding.

**Key Points**

- Longitudinal neuropsychological assessments are feasible in grade 2 glioma patients.
- Quality of life and cognition seem not altered by temozolomide in the short term.
- Patients’ cognitive and emotional functioning should be monitored closely.

**Importance of the Study**

As the expected survival of patients with newly diagnosed diffuse low-grade glioma (DLGG) is long, the possible side effects, particularly on quality of life (QoL) and neuropsychology, must be considered during treatment decision-making. Yet, data on chemotherapy effects on QoL and neuropsychology are scarce in this population. This prospective study assessed the feasibility of a longitudinal QoL, neurocognition, and psychological disorder assessment until disease progression in patients with DLGG receiving first-line temozolomide. The study participation and adherence rates (up to month 12 of treatment) were 89.7% and 95.7%, demonstrating its feasibility. QoL remained stable up to month 6 post-treatment. The results also suggest that self-perceived and objective neurocognitive outcomes remained stable during the follow-up. Temozolomide did not induce psychopathological decompensation. Our study suggests that first-line temozolomide does not worsen QoL and neurocognition in the short term. It also highlights that patients with DLGG often experience neuropsychological burdens that must be managed.

Diffuse low-grade glioma (DLGG; WHO grade II gliomas^1,2^) is a rare tumor affecting mainly people in their thirties or forties. DLGG is characterized by a slow and continuous growth, resulting in few symptoms at diagnosis besides epilepsy and mild cognitive alterations (mostly memory, executive function, and attention).^3,4^ However, DLGG anaplastic transformation is unavoidable. The overall survival ranges from 5 years to >15 years.^5,6^ Maximal safe resection is the first therapeutic option in newly diagnosed patients. Conversely, the timing and choice of chemotherapy or/and radiotherapy (RT) remain controversial. Indeed, due to the patient’s young age, generally preserved quality of life (QoL) at diagnosis and relatively long survival, the management strategy must take into account both the expected oncological benefit of treatments (ie, survival improvement) and the potential short-term and long-term side-effects that can negatively affect patients’ QoL.^7^ In DLGG, neurocognitive functioning alterations can affect the QoL even in the absence of other neurological deficits or intractable epilepsy.^8,9^ In this context, the cognitive impact of tumor resection has been widely evaluated, leading to the generalized use of awake mapping techniques to maximize the extent of resection while preserving cognition.^10^ A possible negative effect of RT on neurocognition has been reported.^11,12^ Therefore, RT is often differed in DLGG patients, in the absence of a survival benefit of early RT.^13^

Temozolomide (TMZ), an orally administered drug, is widely used in DLGG patients,^14–18^ but studies on its effects on neurocognition and QoL are scarce. The available data suggest the absence of negative effects but come from studies with important limitations, precluding any definitive conclusion. Specifically, neurocognitive assessment was performed using a single and questionable test (ie, the Mini-Mental State Evaluation, MMSE), was included in prospective trials as secondary endpoint and/or performed only for a short time during follow-up. Neurocognitive functioning and QoL are affected also by other variables, such as epilepsy and antiepileptic drugs, the premorbid cognition level, and psychological states or disorders (eg, anxiety or depression)^19,20^ that must be taken into account. Moreover, acceptability data on cognitive assessments are scarce in adults with primary brain cancer.^21^ The aim of this study was to evaluate the feasibility of a complete longitudinal assessment (QoL, neurocognition, and psychological disorders) in DLGG patients receiving first-line TMZ.

## Materials and Methods

### Study Design and Objectives

This longitudinal and prospective study in DLGG patients receiving first-line TMZ was performed in a French referral cancer center to evaluate the feasibility (participation and adherence) of a longitudinal evaluation of QoL, neurocognition, and psychological disorders. Feasibility was defined as the completion of these assessments at *Baseline*, months 6 and 12 after TMZ initiation. Patients with tumor progression in the first 12 months after TMZ start were withdrawn from the study in order to prevent cognitive changes due to tumor progression to be falsely attributed to TMZ, as changes in cognitive performances can be observed in relation to tumor progression. The secondary objectives were to describe the QoL and neuropsychological functioning changes during and after TMZ treatment. Patients were screened consecutively over a 2-year inclusion period. No formal statistical sample size calculation was performed.

### Eligibility Criteria

Patients were included based on the following criteria: ≥ 18 years of age; histologically proven DLGG (WHO 2016 classification); first-line TMZ after surgery/biopsy, whatever the delay between the surgical procedure and TMZ initiation; no previous oncological treatment (but for surgery) for the DLGG; ECOG performance status (PS) score ≤ 2; laboratory parameters compatible with TMZ; negative pregnancy test; affiliation to the French social security system. Exclusion criteria were: patients not fluent in French; moderate to severe cognitive impairment (defined as a Montreal Cognitive Assessment [MoCA]^22^ score < 22/30 [Patients screened and found to have a MoCA score over 26 are extremely unlikely to meet clinical and neuropsychological criteria for mild cognitive impairment even after extensive evaluation.^22^ The cutoff of 22, less restrictive than 26, was a good compromise to ensure the validity of the self-reported-outcomes and to not introduce bias in sampling selection.]); visual or auditory deficit (to ensure the validity of the gathered patients-reported-outcomes, in particular, for the paper-and-pencil format questionnaires or for neuropsychological testing that mostly requires visual cognitive processing); known contraindication to TMZ; legal incapacity or physical/psychological/social/geographical condition interfering with the patient’s ability to sign the informed consent or to participate; pregnancy or breastfeeding; unwillingness by patients of childbearing age to use adequate contraception from the study beginning until month 6 after TMZ end; and participation in another clinical trial within 30 days before the study enrollment.

### Study Procedures

Patients were screened during the consultation and included in the study after checking the eligibility criteria. Data on DLGG history and the patient’s clinical status at inclusion were collected. TMZ was given orally according to standard practices (150–200 mg/m^2^/day, from day 1 to 5 of a 28-day cycle). Dose adaptation and treatment duration (up to 24 cycles) were left to the investigator’s discretion based on the clinical and biological tolerance and tumor response (including changes in the FLAIR tumor volume).

The tumor molecular alterations were recorded, including isocitrate dehydrogenase (IDH) mutations (IDH1 R132H immunohistochemistry or direct sequencing of *IDH1* exon 4, +/− *IDH2* exon 4) and 1p19q codeletion (microsatellite analysis for loss of heterozygosity with highly polymorphic markers). Tumor samples were reviewed by an expert neuropathologist (V.R.) to confirm the diagnosis according to the WHO 2016 classification.

Patients were evaluated clinically and by MRI every 3 months during TMZ treatment and the first 24 months after TMZ discontinuation, then every 4–6 months until tumor progression. Progression was defined clinically as a deterioration of neurological symptoms or of the PS score with no other explanation, and/or based on MRI. Radiological progression was defined as an increase of ≥ 25% of the FLAIR tumor volume (manual segmentation of the tumor borders using the AW Server software, version 3.2, General Electrics) compared to *Baseline* or nadir, appearance or progression of an area of contrast enhancement or of a new tumor lesion.

### QoL, Neurocognitive and Psychological Disorder Assessments

QoL, neurocognitive function and psychological disorder assessments were performed by 2 expert clinical neuropsychologists (E.G., L.C.) at inclusion (before TMZ initiation, *Baseline*), then at 6 ± 1 months (*Follow-up 1–FU1*), 12 ± 1 months (*Follow-up 2– FU2*), 18 ± 1 months (if applicable) during TMZ treatment, 6 ± 1 months after TMZ end (*Follow-up 3–FU3*), and then annually (± 1 month) until tumor progression. This manuscript will present data on the assessments performed at *FU1*, *FU2,* and *FU3*.

Each assessment lasted about 3 hours and consisted of (1) a clinical interview (which will be reported elsewhere) and a complete QoL exploration, (2) extensive neurocognitive testing, and (3) psychological evaluation.

(1) QoL was explored using 5 self-reported questionnaires, including the EORTC QoL Questionnaire-Core 30 and its Brain module 20 (QLQ-C30 and QLQ-BN20).^3^ The Multidimensional Fatigue Inventory (MFI-20)^23^ was used to measure fatigue. Cognitive complaints were assessed with the Functional Assessment of Cancer Therapy-Cognitive Function (FACT-Cog) questionnaire.^24^ Memory complaints were explored using the Prospective and Retrospective Memory Questionnaire (PRMQ).^25^ It also included a question on the professional situation.
(2) The neurocognitive assessments were clinician-reported and evaluated (i) the premorbid intellectual ability, using the French National Adult Reading Test (*f*-NART)^26^; (ii) the global neurocognitive functioning, using the MoCA^22^; and (iii) the main neurocognitive functions, detailed in Table 1 and Supplementary Table 1: Working Memory Index, Processing Speed Index, Language, Episodic Memory, Attention, Executive Functions.
(3) Psychological functioning was explored using the Beck Depression Inventory–II (BDI-II),^27^ to measure the presence and severity of depression, the State-Trait Anxiety Inventory (STAI-Y)^28^ for anxiety, and the State-Trait Anger Expression Inventory-II (STAXI-II)^29^ for anger.

**Table 1.** Neuropsychological Tests and the Investigated Neurocognitive Domains

Neuropsychological domain	Measures^a^
**Working memory**	**Working memory index:** Measures the speed and efficiency of memory retrieval processes. *Outcome: Z-score derived*^*48*^ *from the French normative Working Memory Index calculated using the raw scores of the Arithmetic and the Digit span subtests of the WAIS-IV.*
**Processing speed**	**Processing speed index:** Measures the psychomotor speed. *Outcome: Z-score derived*^*48*^ *from the French normative Processing Speed Index calculated using the raw scores of the Coding and the Symbol subtests the WAIS-IV.*
**Language**	**Speech and semantic fluency:** Measures the semantic memory accessibility and availability. *Outcome: Mean Z-score of categoric fluency (the raw scores obtained for the “animal” [easy criteria] and “furniture” [difficult criteria] subtests were aligned to the published French normative Cardebat fluency scores, converted into Z-scores, and averaged).* **Verbal picture denomination** *Outcome*^b^*: DO80 scores*.
**Episodic memory** ^c^	**Hopkins verbal learning test:** Examines the verbal learning capacity and verbal information consolidation into long-term memory (6 alternative versions are available). *Outcomes: Mean Z-scores of learning (the raw number of total trial learning, raw number of total delayed recall and raw discrimination index were aligned to the published French normative data, converted into Z-scores, and averaged).* **Rey-osterrieth complex figure:** Examines the implicit learning capacity of visual short-term memory. *Outcome*^b^*: Immediate recall score*.
**Attention**	**Trail making test:** Measures attention, visual search, and mental control. *Outcome*^b^*: Time score for TMT A completion*. **Bells test:** Assesses spatial neglect, visual search, and processing speed. *Outcome*^b^*: Number of neglected bells*. **Stroop color and word test:** examines information processing speed, selective attention, and mental control. *Outcomes: mean Z-scores of attention (the raw times to complete the Stroop Card A and Stroop Card B were aligned to the published French normative data, converted into Z-scores, and averaged).*
**Executive functioning**	**Literal word fluency task:** assesses verbal initiation and flexibility. *Outcomes: Z-scores of literal fluency [the raw scores obtained for the “p” (easy criteria) and “v” (difficult criteria) subtests were aligned to the published French normative Cardebat fluency scores, converted into Z-scores, and averaged].* **The Kaplan Stroop color-word interference effect:** Measures the capacity to inhibit an over-learned response in favor of an unusual one. *Outcomes: Error*^b^ *scores and slowing on the interference condition of the Stroop Card C (the raw time obtained on the Stroop Card C was aligned to the published French normative data and converted into Z-scores*). **Conceptualization test:** assesses the abstraction abilities. *Outcome: Z-score of abstraction (the raw score obtained on the Similarities subtest of the WAIS-IV was aligned to the French normative data and converted into a Z-score*). **Social cognition tasks:** assesses the cognitive theory of mind that refers to the ability to deduce the mental states of other people (their beliefs and intentions). *Outcomes: mean Z-scores of social cognition [the raw scores of the TOM-15 test (“the cold social cognition”) and the Reading the Mind in the Eyes Test (“the hot social cognition”) were appropriately aligned to the published French normative data, converted into Z-scores and averaged].* **Rey-osterrieth complex figure:** assesses the planning, organizational, visual-spatial, and visual-constructional abilities. *Outcome*^b^*: Type of visual-constructive strategy used to copy the complex figure*. **Concept shifting test:** measures the attention and ability to mentally control simultaneous stimulus patterns and to shift. *Outcomes*^b^*: time scores and shifting errors in the Trail Making Test B*

^a^References are available in Supplementary Table 1

^b^This outcome was not taken into account in the mean Z-score calculation of the main neurocognitive domains shown in Figure 2

^c^Episodic prospective memory also was assessed using a previously described experimental task that will be reported elsewhere.

### Statistical Analyses

All *P* values were 2-sided, with a 5% significance level (*P* < .05). To take into account the multiplicity of tests used in this study, *P*-values were adjusted using the Benjamini and Hochberg methods. Statistical analyses were performed with STATA (version 16.0), SAS (version 9.4), and R (version 4.1.2).

The primary endpoints of this feasibility study were the participation rate (percentage of patients who consented to participate among all screened patients) and the adherence rate (percentage of patients who completed the evaluations at *Baseline*, *FU1* and *FU2* of treatment among the included patients without progression within 12 months ± 40 days after TMZ introduction). It was expected that 80% of the screened patients would consent and that 80% of participating patients would complete the assessments. The expected and observed rates were compared using an exact binomial test.

The QoL and neuropsychological scores were compared according to the relevant French guidelines (Supplementary Table 2). Briefly, scores at the different visits were compared with their baselines. Then, different methods were used to quantify significant changes (deterioration or improvement) based on the available published data: within-group Minimally Important Differences (MID; QLQ-C30), the threshold for changes (QLQ-BN20), 10th percentile to published French normative data (FACT-Cog, TMT, STAI-Y), Reliable Change Index (RCI; PRMQ; Supplementary Table 3), Z-score (Arithmetic, Digit span, Coding, Symbol and Similarities subtests of the Wechsler Adult Intelligence Scale-Fourth Edition [WAIS-IV], Cardebat fluency test, Hopkins Verbal Learning Test [HVLT], Stroop Kaplan test, TOM-15 test, Reading the Mind in the Eyes Test and STAXI-II), or relevant cutoff score (DO80, Bells Test, Rey-Osterrieth Complex Figure, and BDI-II).

### Ethical and Regulatory Considerations

The study was conducted in accordance with the Good Clinical Practice guidelines as defined by the International Conference on Harmonization, STROBE guidelines, and the French regulations in force. It was registered at ClinicalTrials.gov (NCT03257618) and approved by an Ethics Committee (#AU1334; 2017-001108-31; 12/06/2017). The patient’s written informed consent was obtained before any procedures.

## Results

### Participation Rate and Population Included in the Study

**Figure 1.**
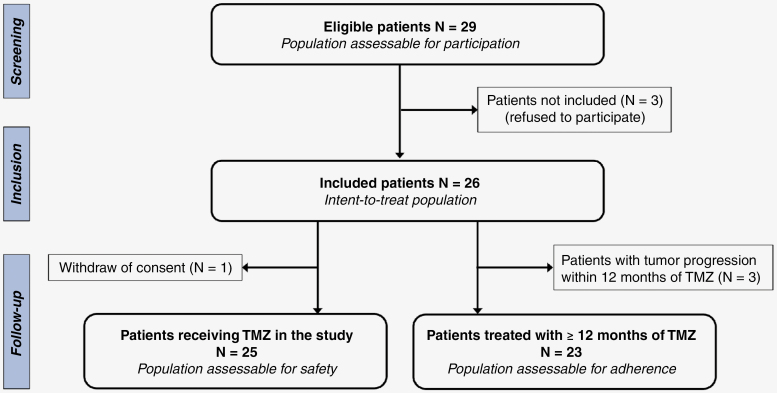
Study flow-chart.

Among the 29 eligible patients, 26 accepted to participate and were included in the study between July 2017 and March 2020 (Figure 1). Of note, no patient was deemed noneligible due to a moderate or severe cognitive impairment, or to a visual/auditory deficit. The participation rate was 89.7% (95% CI: 72.6%–97.8%), which did not significantly differ from our hypothesis of a participation rate of 80% (*P* = .25). The main characteristics of patients are detailed in Table 2. At inclusion, the median age was 45 years (range 29–73), all patients had a PS score of 0–1 and 61.5% were still working. The median sociocultural level^30^ was 3 (ie, moderate to high). Moreover, 46.1% of patients reported epileptic seizures (mostly partial) and 69.2% had no neurological deficit (besides cognitive disturbances; sensitive and/or motor deficit in 7.7%). At inclusion, 80.8% of patients received antiepileptic drugs and 7.7% psychotropic drugs (but none corticosteroids); 19.2% received speech therapy, 19.2% physical therapy, and 19.2% psychotherapy. The median FLAIR tumor volume was 18.6 cm^3^ (range 2.5–38.8) and no contrast enhancement was detected after gadolinium injection.

**Table 2.** Main Clinical, Biological, and Radiological Characteristics of the Patients With Diffuse Low-Grade Glioma at TMZ Initiation

Sex	
Male, *n* (%)	15 (57.7)
Female, *n* (%)	11 (42.3)
Tumor side	
Right hemisphere, *n* (%)	6 (23.1)
Left hemisphere, *n* (%)	20 (76.9)
Tumor location	
Frontal, *n* (%)	4 (15.4)
Temporal +/− insular, *n* (%)	5 (19.2)
Fronto-insular, *n* (%)	5 (19.2)
Fronto-temporo-insular, *n* (%)	6 (23.1)
Parietal, *n* (%)	6 (23.1)
*Histological-molecular diagnosis (WHO 2016)*	
Oligodendroglioma, mutant IDH and 1p19q codeled, *n* (%)	12 (46.1)
Diffuse astrocytoma, mutant IDH, *n* (%)	13 (50.0)
Diffuse astrocytoma, wild-type IDH, *n* (%)	1 (3.8)
Foci of malignant transformation within an otherwise grade 2 tumor, *n* (%)	2 (7.7)
*Number of previous surgeries*	
1, *n* (%)	21 (80.8)
2, *n* (%)	4 (15.4)
3, *n* (%)	1 (3.8)
*Surgery type before TMZ initiation (awake in 25/26 patients)*	
Subtotal, *n* (%)	25 (96.1)
Partial, *n* (%)	1 (3.8)
In awake conditions, *n* (%)	25 (96.1)
Median age in years at baseline (range)	45 (29–73)
Median time after last surgery in months (range)	36 (1–96)
Median time after diagnosis in months (range)	47 (4–121)
*Personal situation*	
Single, widow or divorced *n* (%)	5 (19.2)
Married/cohabiting, *n* (%)	21 (80.8)
*Professional situation*	
Full-time work, *n* (%)	10 (38.5)
Part-time work, *n* (%)	6 (23.1)
Permanent disability, *n* (%)	6 (23.1)
Sick leave, *n* (%)	1 (3.8)
No activity, *n* (%)	1 (3.8)
*Sociocultural level*	
Years of study, median (range)	14 (8–22)
*Sociocultural level,*^*30*^ *n (%)*	
2	3 (11.5)
3	14 (53.8)
4	9 (34.6)
*ECOG performance status score*	
0, *n* (%)	15 (57.7)
1, *n* (%)	11 (42.3)
*Epileptic seizures*	
Any type, *n* (%)	12 (46.2)
Partial, *n* (%)	11 (42.3)
Generalized, *n* (%)	2 (7.7)
Median tumor volume (cm^3^) on FLAIR-weighted sequence (range)	18.6 (2.5–38.8)

FLAIR, fluid attenuation inversion recovery.

### TMZ Treatment and Tumor Response

As one patient withdrew consent shortly after inclusion in the study, data on TMZ treatment and safety were collected from 25 patients. The median number of TMZ cycles was 12 (range 9–23), corresponding to a median treatment duration of 13 months (range 8–22). The dose was reduced and/or treatment was temporarily discontinued in 15 patients (60.0%) due to hematological toxicity (*N* = 10), intercurrent viral infection (*N* = 2), weight loss (*N* = 1), and personal convenience (*N* = 1). Grade 3 or 4 toxicity events occurred in 6 patients (24.0%; Supplementary Table 4). TMZ was discontinued due to optimal tumor response or stability in 20 patients (80.0%). Among 12 patients who presented with seizures at the time of TMZ initiation, 4 (33.3%) were seizure-free at FU1 while 8 (66.7%) still had partial seizures, of which 6 patients (75.0%) became seizure-free at FU2. One patient (who had partial seizures at the first symptom of the tumor) was seizure-free at TMZ initiation but presented with partial seizures again at FU1, FU2, and FU3, with a stable frequency (3–4 per month). Tumor progression during treatment was observed in 5 patients (after 9 cycles for 3 patients and after 18 cycles for 2 patients), defined radiologically as an increase of the FLAIR tumor volume with the appearance of contrast enhancement in 3/5 patients.

### Adherence to the Longitudinal Assessment of QoL and Neuropsychology

Adherence to the longitudinal assessment could be evaluated in 23/26 patients due to tumor progression in 3 patients within 12 months following TMZ initiation (Figure 1). One patient withdrew consent shortly after inclusion in the study and 22 completed the complete neuropsychological evaluations at *Baseline*, *FU1* and *FU2*. This corresponded to an adherence rate of 95.7% (95% CI: 78.1–99.9) that was not different from that of our initial hypothesis (80%, *P* = .07).

The *FU1*, *FU2,* and *FU3* visits were performed after a median of 6 months (range 5–7), 11 months (range 11–13), and 7 months (range 6–12), respectively. Deviations ≥ 1 month were mostly due to the COVID-19 pandemic impact.

### QoL Changes

The changes in the QLQ-C30 scores are shown in Supplementary Figure 1 and Table 5. Based on the within-group MIDs (Table 3), the scores of the functional scales “Global health status/QoL” and “Cognitive functioning” and of the symptom scale “Fatigue” remained stable at all 3 visits. The ‘Emotional functioning’ scale score improved at *FU1* and *FU2*, then remained stable at *FU3*. Conversely, QoL deteriorated transitorily at *FU1* regarding the functional scale ‘Social functioning’ (ie, diminished scores) and the symptom scale ‘Nausea/vomiting’ (ie, increased scores).

**Table 3.**
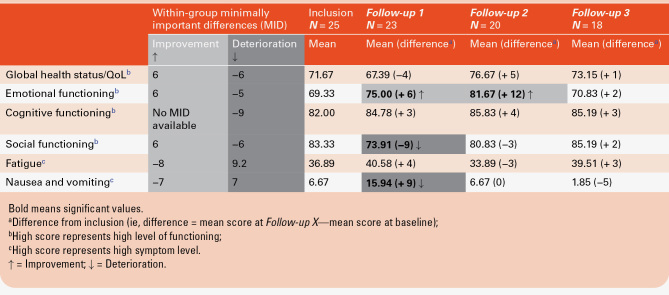
Changes in Quality of Life, Assessed With 4 Functional and 2 Symptom Scales of the EORTC QLQ-C30 Questionnaire, Over Time

The changes in the QLQ-BN20 scores are in Supplementary Figure 2 and Table 5. Only the ‘Headache’ score tended to change during the follow-up (*P* = .08). Based on the 10-point change threshold, 4 symptom scales of interest were analyzed (Table 4). Compared with *Baseline*, the ‘Headache’ scores remained stable or improved in 95.5%, 94.8%, and 100% of patients at *FU1, FU2,* and *FU3*, respectively. The ‘Future uncertainty’ scores remained stable or improved in 81.8%, 84.2%, and 82.4% of patients, at *FU1, FU2,* and *FU3*, respectively. The ‘Seizures’ scores remained stable or improved in 90.3%, 100%, and 94.1% of patients at *FU1, FU2,* and *FU3*. Conversely, the ‘Communication deficit’ scores worsened in 40.9%, 26.3%, and 23.5% of patients at *FU1, FU2,* and *FU3*, compared with *Baseline*.

**Table 4.** Overall Changes, Compared With *Baseline*, in Disease-Specific Health-Related Quality of Life, Assessed With 4 Symptom Scales of the EORTC QLQ-BN20 Questionnaire, Over Time

	*Follow-up visit*		
	*Follow-up 1*	*Follow-up 2*	*Follow-up 3*
*Symptom scales*	*N* = 26	*N* = 23	*N* = 21
*Headache*			
Decline ^a^	1/22 (4.6%)	1/19 (5.2%)	0 / 17 (0.0%)
Improvement ^b^	10/22 (45.5%)	6/19 (31.6%)	8 /17 (47.1%)
Stable ^c^	11/22 (50.0%)	12/19 (63.2%)	9/17 (52.9%)
Missing data	4	4	4
*Future uncertainty*			
Decline ^a^	4/22 (18.2%)	3/19 (15.8%)	3/17 (17.6%)
Improvement ^b^	5/22 (22.7%)	6/19 (31.6%)	7/17 (41.2%)
Stable ^c^	13/22 (59.1%)	10/19 (52.6%)	7/17 (41.2%)
Missing data	4	4	4
*Seizures*			
Decline ^a^	2/22 (9.1%)	0/19 (0.0%)	1/17 (5.9%)
Improvement ^b^	2/22 (9.1%)	3/19 (15.8%)	2/17 (11.8%)
Stable ^c^	18/22 (81.2%)	16/19 (84.2%)	14/17 (82.3%)
Missing data	4	4	4
*Communication deficit*			
Decline ^a^	9/22 (40.9%)	5/19 (26.3%)	4/17 (23.5%)
Improvement ^b^	7/22 (31.8%)	5/19 (26.3%)	6/17 (35.3%)
Stable ^c^	6/22 (27.3%)	9/19 (47.4%)	7/17(41.2%)
Missing data	4	4	4

^a^Decline = Score increase of ≥ 10 points compared with baseline;

^b^Improvement = Score decrease of ≥ 10 points compared with baseline;

^c^Stable = Score change < 10 points compared with baseline.

The MFI-20 scores were not significantly different among visits (*P* = .82 for the total; *P* > .05 for the different dimensions; Supplementary Table 5).

The FACT-Cog scores remained stable over time (*P* > .05; Supplementary Table 5). Specifically, the ‘Perceived Cognitive Impairment’ subscale score was significantly higher than the normative score in 38.5% of patients (*n* = 10/26) at *Baseline*, 50% (*n* = 12/24) at *FU1*, 50% (*n* = 11/22) at *FU2*, and 52.6% (*n* = 10/19) at *FU3*. The ‘Impact on Quality of Life’ subscale score was significantly higher than the normative score in 38.5% of patients (*n* = 10/26), 33.3% (*n* = 8/24), 18.2% (*n* = 4/22), and 15.9% (*n* = 3/19), respectively.

Concerning memory complaints, the prospective (*P* = .90) and retrospective memory scores (*P* = .48) measured with the PRMQ were not significantly different among visits (Supplementary Table 5). Using the RCI (Supplementary Table 3), the Retrospective Memory subscale score was stable in 100% of patients among visits. The Prospective Memory subscale score remained stable or decreased (ie, fewer prospective memory errors in daily life) in 100%, 94.1 %, and 86.7% of patients at *FU1*, *FU2,* and *FU3*, respectively.

Lastly, the percentage of patients who continued working did not differ among visits: 61.5%, 57.7%, 60.9%, and 57.1%, respectively. Compared with *Baseline*, 2 patients increased their work hours at *FU3*, whereas 2 patients had to reduce their professional activity (sick leave and permanent disability).

Because of the limited number of patients with epileptic seizures at TMZ initiation, correlations between epileptic activity and QoL were not analyzed, but patients reported no significant changes in terms of QoL using the subjective BN20 questionnaire (Supplementary Table 4).

### Changes of Neurocognition

At the *Baseline*, the median *f*-NART score was 28 (range 11–35), and the premorbid Intellectual Quotient was (above) normal (median 110, range 85–119) in 92.3% of patients. The MoCA scores did not significantly change during the follow-up (*P* = .20).

**Figure 2.**
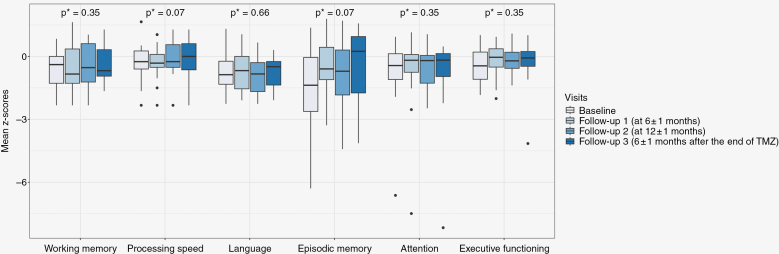
Boxplots showing the mean Z-scores of the neurocognitive tests to assess the main neurocognitive domains during the follow-up (*P* values obtained using the Friedman test).

The changes in the mean Z-scores of the different neurocognitive function tests at the different visits are presented in Figure 2 and Supplementary Table 6. The mean Z-scores for Episodic Memory and Processing Speed tended to increase over time (*P* = .07 for both). The mean Z-scores of Working Memory tended to decrease over time (*P* = .35), whereas the mean Z-scores of Language (*P* = .66), Attention and Executive functions (both *P* = .35) tended to improve. The DO80 (*P* = .90), Trail Making Test A and B (*P* = .98 and *P* = .87) and Bells test (*P* = .65) scores did not differ over time (Supplementary Figure 3).

### Changes in Psychopathology

The BDI-II, STAI-Y, and STAXI-II scores did not significantly change among visits (*P* = .46, *P* = ,17, *P* = .79, respectively; Supplementary Table 7). Similarly, the percentage of patients with a pathological score (Supplementary Figure 4) and/or who met the criteria for major depressive/anxiety/anger disorder did not change significantly over time (*P* > .05).

At *Baseline*, 42.3% of patients (*n* = 11/26) received supportive care (ie, speech/neurocognitive/physiological/psychotherapy), decreasing to 36% (*n* = 9/25) at *FU1*, 27.3% (*n* = 6/22) at *FU2*, and 21.1% (*n* = 4/19) at *FU3*. Psychological support concerned 5, 7, 3, and 2 patients at *Baseline*, *FU1*, *FU2*, and *FU3*, respectively.

## Discussion

### Feasibility

This study demonstrated the feasibility of a longitudinal assessment of QoL, neurocognition, and psychological disorders in patients with DLGG (participation rate 89.7%, adherence rate 95.6%). Adherence was high despite the extensive, time-consuming assessment, as well as organizational difficulties due to the COVID-19 pandemic. In comparison, in the EORTC 22 033 phase 3 study that compared TMZ and RT in patients with DLGG, compliance for the QoL evaluation was 67% in both arms at month 12 (vs. 91% in our study) and 58% at month 36. Compliance for the cognitive functioning assessment (MMSE) was 63.7% at month 12 and 52.0% at month 36^31^ and for the memory functioning evaluation was 82.6% at month 6 and 73.9% at month 12 after the *Baseline* visit.^32^ One reason for the high level of adherence we observed could be that despite the observational nature of our study, patients may have indirectly benefited from these repeated evaluations. In our study, each assessment lasted about 3 hours and consisted of a clinical interview and a complete QoL exploration, an extensive neurocognitive testing, and a psychological evaluation. Assessments were conducted by an expert clinical neuropsychologist: No feedback nor help was given to the patients while undergoing an assessment of these issues with scales and questionnaires. However, the clinical interviews gave the patients the opportunity to express their neuropsychological and QoL complaints “with their own words,” to have their emotions addressed with empathetic responses and respect, and to receive an appropriate response from a trained clinical psychologist. The benefits produced by these aspects of the relationships (including a relationship with a health caregiver and expectations in the effectiveness of the treatment) are smaller than those produced by active psychotherapies, but they are effective (and generally named “placebo effects”). Therefore, the longitudinal follow-up of patients in our study could be considered as a non-pharmacological intervention. In this field, the adherence rate for optimum therapeutic efficacy has historically been set at 80%.^33,34^ Overall, the feasibility of our evaluation of neuropsychology and QoL in patients with DLGG receiving TMZ can be considered high.

### Limited Effect of TMZ on QoL, Neuropsychology in the Short-Term

The treatment timing and choice following DLGG resection are still debated and must take into account various clinical, biological, and radiological parameters. In any case, the short- and long-term effects of treatments on QoL and neurocognitive functioning must be assessed. Indeed, several ongoing prospective trials are evaluating composite primary outcomes that include the preservation of QoL and/or neurocognition (eg, NCT02444000 and NCT04702581 trials: “survival without neurocognitive deterioration”; NCT05331521 trial “qualified overall survival”). Yet, data on the impact of treatments on QoL and neurocognition are scarce in most phase 2 and 3 trials, with frequent adherence issues and limited cognition assessment.^21,31,32,35^

Temozolomide is frequently proposed as a first-line treatment in patients with DLGG requiring treatment after surgical resection.^15^ Its impact on QoL in the short-term is expected to be low, because of its limited toxicity. No disruption of treatment due to toxicity was observed in our study. At all follow-up visits, the scores of the functional scales “Global health status/QoL” and “Cognitive functioning” remained stable (or improved), as well as those of the symptom scales “Fatigue,” “Headache, ”Future uncertainty’ and Seizures’. The “Social functioning” and the “Nausea/vomiting” scale scores worsened transiently, in line with previous studies.^31^ Overall, our data suggest a limited effect of TMZ on QoL and neurocognition in the short term, consistent with the few published studies. In the EORTC 22033 phase 3 trial (RT vs. TMZ in 237 “high-risk” DLGG patients), QoL (QLQ-C30 and BN20) and global cognitive functioning (MMSE) were not worsened at month 36 after TMZ initiation. However, the adherence rates to the assessments were only 58% and 50%, respectively.^31,32^ In our study, global cognitive functioning remained stable over time, using the MoCA, a more sensitive test than the MMSE in patients with brain tumors.^36^ In the 22033 EORTC trial, no effect of TMZ (12 months) was observed on the memory functioning of 46 patients, using the Visual Verbal Learning Test (HTLV).^32^ In our study, episodic memory (HVLT) tended to improve over time. Besides the EORTC 22033 trial, the few other studies on the impact of TMZ^37–40^ suggest that QoL is preserved in DLGG patients on TMZ, despite some methodological issues. Specifically, these studies only reported short-term evaluations. Interesting long-term QoL outcomes have been reported.^3,41,42^ However, these studies do not provide any additional information on the effect of each treatment modality because patients were treated with multiple lines of treatment at the time of evaluation.

Fatigue negatively influences cognitive complaints and is a frequent issue in DLGG patients.^43^ In line with the results of the EORTC 22033 trial^14^ and a previous retrospective study in 25 DLGG patients receiving TMZ,^44^ of our patients did not report fatigue worsening during TMZ treatment. Moreover, the percentage of patients still working on TMZ did not significantly change over time.^45^

The percentage of patients with psychological disorders did not change over time. This suggests that TMZ, combined with adapted supportive care, does not promote psychiatric or psychological decompensation. Previous studies showed that patients with DLGG frequently report mood/emotional disorders, particularly depression and/or anxiety.^20,46,47^ In our study, psychological distress was common before chemotherapy: 26.9% of patients reported moderate or elevated anxiety, 38.5% severe depressive symptoms, and 3.4% severe anger. This highlights the emotional burden of DLGG patients that must be managed.

### Subjective and Objective Complaints

Overall, in our study, cognitive and memory complaints did not increase during/after TMZ treatment. As subjective complaints are influenced by objective neurocognitive functioning, fatigue, and psychopathological variables, they seem to be an interesting measure of the patient’s QoL. Yet, only one study included a subjective cognitive assessment among the 21 studies on the feasibility and acceptability of cognitive functional assessments in patients with brain tumors.^21^ In this context, the trend towards an improvement of episodic memory is interesting. This finding cannot only be explained by a memory bias (“retest effect”) because alternative test versions were used at the different follow-up visits. Moreover, in our small sample, memory complaints remained stable over time. Interestingly, the reported frequency of prospective memory errors was stable or decreased in most patients over time. This raises the question of whether the improvement in memory encoding efficiency (measured by the HVLT) could have been transferred into the patient’s daily life, particularly in the tasks involving prospective memory. This is plausible because psychological distress remained stable over time, and processing speed tended to improve.

Processing speed is a measure of the time required to respond to and/or process information in an ongoing environment.^48^ Many studies demonstrated that slower information processing speed inevitably affects memory, decision-making, and other neurocognitive functions. In patients with temporal glioma, processing speed was predictive of social well-being outcomes.^47^ In our sample, the mean Z-score of processing speed tended to increase over time, particularly at *FU3*. In parallel, the social QoL deterioration objectified at *FU1* was reversed at *FU3*. This observation can be added to the growing evidence that neurocognitive impairment and QoL deficits are related, and their relationships may change dynamically during the disease course and TMZ treatment.

### Strengths and Limitations

To the best of our knowledge, this is the first longitudinal study on the feasibility and acceptability of extensive QoL, neurocognitive, and psychological assessments of DLGG patients treated with first-line TMZ. Of note, one patient in our study had an IDH wild-type tumor, an entity that does not qualify as a DLGG using the latest 2021 WHO classification. Indeed, when the study was designed and the inclusions started (2017), the WHO classification in force was the 2016 classification. We considered that retrospectively modifying the eligibility criteria would not be methodologically correct. Also, we hypothesize that the impact of TMZ on QoL or cognition does not depend a priori on the IDH status.

As the number of patients to be included was calculated to address the primary objective of feasibility, our study was not powered to address changes in the QoL or neuropsychological functioning on TMZ. Therefore, the results of the longitudinal assessments were analyzed as exploratory measures and must be considered with caution. However, the large number of repeated measurements obtained in the study may be a strength. Moreover, due to the limited number of patients and the multiplicity of tests, we adjusted all *p*-values with the Benjamini and Hochberg method, a more powerful procedure than the Bonferroni correction to adjust for false discovery rate.

Because the aim was to evaluate the feasibility of longitudinal assessments, the study cohort was not compared to a control group, which may limit the clinical relevance of findings. Given that cognition is impacted by a number of variables in patients with DLGGs (eg, premorbid level, tumor location, surgery, epilepsy and antiepileptic drugs, psychological functioning, etc.), comparing the performances of patients on TMZ with their own performances before TMZ may be the most valid method to assess the impact of TMZ on cognition and QoL. Indeed, comparing patients receiving TMZ to a control group would require matching cases and controls on all the cited variables, which is not feasible.

Another limitation is that we reported only short-term results, although an evaluation of the longer-term effect of TMZ is crucial to guide treatment decision-making in DLGG.^46^ As patients without tumor progression are still followed, long-term data will be reported later.

More accurate tools to evaluate QoL in DLGG patients are needed. We chose the widely used EORTC-QLQ-C30 and BN20 questionnaires. Yet, these tools do not seem completely appropriate for these long-surviving patients. Some symptoms (eg, visual disturbances, motor deficit, bladder dysfunction) are infrequent in patients with DLGG receiving first-line treatment, while important issues are not considered (eg, professional/career limitations, loan/insurance restrictions). Indeed, contrarily to other brain tumors (such as glioblastomas), only a few patients with DLGG suffer from permanent neurological deficits during the early phase of their disease (except from neurocognitive alterations): no patient was excluded from the study for this reason. Moreover, patients are asked to evaluate their symptoms “during the last week.” As follow-up visits were scheduled during the pause of TMZ, short-term changes related to TMZ intake might not have been captured. Qualitative data from the interviews may allow us to apprehend more adequately TMZ-related symptoms.

Lastly, a crucial issue when analyzing longitudinal data is the lack of homogeneous definition of the changes that are clinically relevant and not only statistically significant. As QoL deterioration and neurocognitive impairment in DLGG can be linked also to the tumor, surgery, epilepsy, antiepileptic drugs, and depression,^49^ we chose to compare the patients’ performances to their own baseline score. To this end, it is crucial to define, for each QoL or neuropsychological score, what is a clinically meaningful change. We used different methods in function of the available published data (eg, MID, RCI, cutoff, point-threshold). Efforts have been made to define such changes for the EORTC questionnaires^50^; however, thresholds to define clinically relevant changes are still discussed. The same issue concerns also the scores used for the neurocognitive assessments. Z-scores and percentile ranks, when available, allow for comparing the patients’ performance to that of their matched controls. However, a change in a Z-score/rank does not systematically reflect a change in daily life functioning. Yet, patient-reported outcomes, with subjective self-perceived neurocognition assessments, are often lacking.^21^ Our study confirms that they may provide a crucial understanding of the patients’ neuropsychological changes to offer them adapted and appropriate supportive care.

## Conclusion

In this prospective study, DLGG patients receiving first-line TMZ underwent an extensive longitudinal assessment of QoL, neurocognition, and psychological disorders by an expert clinical neuropsychologist. Despite some limitations and methodological issues (particularly, the clinically relevant changes), our data show that completing such an assessment during treatment is feasible, as indicated by the high participation and compliance rates. In the absence of curative treatment for DLGG and considering the long-term survival of these patients, preserving QoL, neurocognition and mood is a major goal. The importance of assessing these outcomes when evaluating treatment strategies (including new systemic agents and RT) is now widely accepted. Our results suggest that TMZ has a low effect on QoL and neuropsychology in the short term. These findings now need to be confirmed during the long-term follow-up, in a larger population.

## Supplementary Material

vdae084_suppl_Supplementary_Tables_1

vdae084_suppl_Supplementary_Tables_2

vdae084_suppl_Supplementary_Tables_3

vdae084_suppl_Supplementary_Tables_4

vdae084_suppl_Supplementary_Figures_1

vdae084_suppl_Supplementary_Figures_2

vdae084_suppl_Supplementary_Tables_5

vdae084_suppl_Supplementary_Tables_6

vdae084_suppl_Supplementary_Figures_3

vdae084_suppl_Supplementary_Tables_7

vdae084_suppl_Supplementary_Figures_4

## Data Availability

Anonymized data not published within this article will be made available by request from any qualified investigator.
