# Supplementary material for: Longitudinal assessment of quality of life, neurocognition, and psychopathology in patients with low-grade glioma on first-line temozolomide: A feasibility study
Source: Neurooncol Adv. 2024 Jun 4;6(1):vdae084. doi: 10.1093/noajnl/vdae084 (PMC11212068; doi:10.1093/noajnl/vdae084)
Supplement: vdae084_suppl_Supplementary_Tables_1 [file vdae084_suppl_Supplementary_Tables_1.docx]

**Supplementary Table 1.** References of the neuropsychological tests used in the study.

| **Neuropsychological domain** | **Test** | **References** |
| --- | --- | --- |
| **Working Memory** | **Subtests of the WAIS-IV: Arithmetic**  **Digit span** | Wechsler D. *WAIS-IV Nouvelle Version de l’échelle d’intelligence de Wechsler Pour Adultes, Quatrième Édition*. Edition du Centre de Psychologie Appliquée; 2011. |
| **Processing Speed** | **Subtests of the WAIS-IV:**  **Coding**  **Symbol** | Wechsler D. *WAIS-IV Nouvelle Version de l’échelle d’intelligence de Wechsler Pour Adultes, Quatrième Édition*. Edition du Centre de Psychologie Appliquée; 2011. |
| **Language** | **Cardebat fluency**  **DO80** | Cardebat D, Doyon B, Puel M, Goulet P, Joanette Y. [Formal and semantic lexical evocation in normal subjects. Performance and dynamics of production as a function of sex, age and educational level]. *Acta Neurol Belg*. 1990;90(4):207-217.  Deloche G, Hannequin D. *Test de dénomination orale d’images: DO 80*. ECPA, Les éditions du Centre de psychologie appliquée; 1997. |
| **Episodic memory^c^** | **Hopkins Verbal Learning Test**  **PROMESSE**  **Rey-Osterrieth Complex Figure** | Rieu D, Bachoud-Lévi AC, Laurent A, Jurion E, Dalla Barba G. [French adaptation of the Hopkins Verbal Learning Test]. *Rev Neurol (Paris)*. 2006;162(6-7):721-728. doi:10.1016/s0035-3787(06)75069-x  Guerdoux-Ninot E, Bauchet L, Legninda Sop FY, et al. Prospective memory and brain metastases: a relevant target for rehabilitation in post-operative patients? *J Neurooncol*. 2020; 147(1):185-194. doi:10.1007/s11060-020-03414-x  Rey A. *Test de Copie d’une Figure Complexe*. Éditions du centre de psychologie appliquée; 1959. |
| **Attention** | **Trail making test A**  **Bells test**  **Kaplan Stroop test A and B** | Ashendorf L, Jefferson AL, O’Connor MK, Chaisson C, Green RC, Stern RA. Trail Making Test errors in normal aging, mild cognitive impairment, and dementia. *Arch Clin Neuropsychol*. 2008; 23(2):129-137. doi:10.1016/j.acn.2007.11.005  Rousseaux M, Beis JM, Pradat-Diehl P, et al. [Presenting a battery for assessing spatial neglect. Norms and effects of age, educational level, sex, hand and laterality]. *Rev Neurol (Paris)*. 2001;157(11 Pt 1):1385-1400.  Godefroy O, Azouvi P, Robert P, et al. Dysexecutive syndrome: Diagnostic criteria and validation study. *Ann Neurol*. 2010; 68(6):855-864. doi:10.1002/ana.22117 |
| **Executive functioning** | **Cardebat fluency**  **Kaplan Stroop test C**  **Similarities of the WAIS-IV**  **TOM-15 test**  **Rey-Osterrieth Complex**  **Trail Making Test B**  **Reading the Mind in the Eyes Test** | Cardebat D, Doyon B, Puel M, Goulet P, Joanette Y. [Formal and semantic lexical evocation in normal subjects. Performance and dynamics of production as a function of sex, age and educational level]. *Acta Neurol Belg*. 1990; 90(4):207-217.  Godefroy O, Azouvi P, Robert P, et al. Dysexecutive syndrome: Diagnostic criteria and validation study. *Ann Neurol*. 2010;68(6):855-864.  doi:10.1002/ana.22117  Wechsler D. *WAIS-IV Nouvelle Version de l’échelle d’intelligence de Wechsler Pour Adultes, Quatrième Édition*. Edition du Centre de Psychologie Appliquée; 2011.  Desgranges B, Laisney M, Bon L, et al. TOM-15: Une épreuve de fausses croyances pour évaluer la théorie de l’esprit cognitive. *Rev Neuropsychol*. 2012;4(3):216-220.  Rey A. *Test de Copie d’une Figure Complexe*. Éditions du centre de psychologie appliquée; 1959.  Ashendorf L, Jefferson AL, O’Connor MK, Chaisson C, Green RC, Stern RA. Trail Making Test errors in normal aging, mild cognitive impairment, and dementia. *Arch Clin Neuropsychol*. 2008; 23(2):129-137. doi:10.1016/j.acn.2007.11.005  Bertoux M, Delavest M, de Souza LC, et al. Social Cognition and Emotional Assessment differentiates frontotemporal dementia from depression. *J Neurol Neurosurg Psychiatry*. 2012;83(4):411-416. doi:10.1136/jnnp-2011-301849 |
