# Supplementary material for: Longitudinal assessment of quality of life, neurocognition, and psychopathology in patients with low-grade glioma on first-line temozolomide: A feasibility study
Source: Neurooncol Adv. 2024 Jun 4;6(1):vdae084. doi: 10.1093/noajnl/vdae084 (PMC11212068; doi:10.1093/noajnl/vdae084)
Supplement: vdae084_suppl_Supplementary_Tables_2 [file vdae084_suppl_Supplementary_Tables_2.docx]

**Supplementary Table 2.** Additional information on the methodology and statistical analyses regarding the QoL, neurocognitive and psychological disorder data.

| **Data from** | **Methodology and statistical analyses used to define changes** |
| --- | --- |
| **QoL questionnaires :** | |
| **EORTC QLQ-C30** | Its scales were scored according to the EORTC recommendations. Only patients with data at baseline were included in the analyses. As in previous published works^1^, we focused on four functional scales (‘Global health status/QoL’, ‘Emotional functioning’, ‘Cognitive functioning’, ‘Social functioning’) and two symptom scales (‘Fatigue’, ‘Nausea/Vomiting’). The within-group minimally important differences (MID) reported by Dirven et al.^2^ was used to quantify the presence of significant deterioration or improvement in their scores at the follow-up visits compared with baseline. For sub-dimensions with multiple thresholds, the most restrictive threshold was selected. |
| **EORTC BN-20** | For the symptom scales ‘Future uncertainty’, ‘Communication deficit’, ‘Headache’ and ‘Seizures’, the status of each patient was classified as improved, stable or deteriorated according to the 10-point change threshold for changes in QLQ-BN20 scores compared with baseline^3,4^. |
| **MFI-20** | Was scored according to the recommendations for the validated French version, with four dimensions compared with five in the English version^5^. Score differences over time were compared using the Friedman test. |
| **FACT-Cog** | Was scored according to the French FACIT recommendations ^6^. Patients with scores ≤10^th^ percentile relative to the normative data ^7^ were considered as having significative complaints (*i.e.*, cognitive complaints higher than matched controls). Changes in the percentage of patients with significative complaints over time were described for the subscales ‘Perceived Cognitive Impairment’ (PCI) and ‘Impact on Quality of Life’ (IQoL). |
| **PMRQ** | Relevant PRMQ score changes were identified compared with baseline using the Reliable Change Index (RCI)^8^ and estimates from a previous study^9^. |
| **Neurocognitive tests :** | |
| **MoCA** | The cut-off was set at 26 (*i.e.,* scores ≤ 25 indicated impairment, with correction for the education level effect by adding 1 point to the total score for patients with ≤12 years of education)^10^. |
| **Subtests of the WAIS-IV (Arithmetic, Digit Span, Coding, Symbol, Similarities)**  **Cardebat fluency**  **HVLT**  **Kaplan Stroop test**  **RMET** | According to the accepted neuropsychological practice^11,12^, was defined as a clinically Pathological (P) an individual Z-score of -1.65 standard deviation below the mean of controls on a domain, or situated at or below the 5^th^ percentile; as Borderline (B) a Z-score between -1.65 and -1, or between the 5^th^ and the 15^th^ percentile; and as Normal (N) a Z-score ≥1 (or > 15^th^ percentile). To this end, all patients’ raw scores were aligned to the relevant published French normative data (adjusted for socio-cultural level, age, and sex) and subsequently converted into Z-scores. |
| **TMT A and B** | Scores were aligned to the percentile rank. |
| **DO80**  **Rey-Osterrieth**  **Complex Figure**  **Bells test** | Scores were compared with a simple cut-off score (adjusted for socio-cultural level, age, and sex). |
| **Psychological scales :** | |
| **BDI-II**  **STAI-Y**  **STAXI-II** | They were scored according to the relevant French guidelines. Raw scores were converted into the published French cut-off scores (adjusted for age for the BDI-II), into T-scores and aligned to the percentile rank (STAI-Y) or into the published French normative data and then converted into Z-scores (STAXI-II). |

1. Reijneveld JC, Taphoorn MJB, Coens C, et al. Health-related quality of life in patients with high-risk low-grade glioma (EORTC 22033-26033): a randomised, open-label, phase 3 intergroup study. *Lancet Oncol*. 2016;17(11):1533-1542. doi:10.1016/S1470-2045(16)30305-9

2. Dirven L, Musoro JZ, Coens C, et al. Establishing anchor-based minimally important differences for the EORTC QLQ-C30 in glioma patients. *Neuro-Oncol*. 2021;23(8):1327-1336. doi:10.1093/neuonc/noab037

3. Maringwa J, Quinten C, King M, et al. Minimal clinically meaningful differences for the EORTC QLQ-C30 and EORTC QLQ-BN20 scales in brain cancer patients. *Ann Oncol*. 2011;22(9):2107-2112. doi:10.1093/annonc/mdq726

4. Wong E, Zhang L, Kerba M, et al. Minimal clinically important differences in the EORTC QLQ-BN20 in patients with brain metastases. *Support Care Cancer*. 2015;23(9):2731-2737. doi:10.1007/s00520-015-2637-5

5. Gentile S, Delaroziere JC, Favre F, Sambuc R, San Marco JL. Validation of the French ‘multidimensional fatigue inventory’(MFI 20). *Eur J Cancer Care (Engl)*. 2003;12(1):58-64.

6. Joly F, Lange M, Rigal O, et al. French version of the Functional Assessment of Cancer Therapy–Cognitive Function (FACT-Cog) version 3. *Support Care Cancer*. 2012;20(12):3297-3305. doi:10.1007/s00520-012-1439-2

7. Lange M, Heutte N, Morel N, Eustache F, Joly F, Giffard B. Cognitive complaints in cancer: The French version of the Functional Assessment of Cancer Therapy–Cognitive Function (FACT-Cog), normative data from a healthy population. *Neuropsychol Rehabil*. 2016;26(3):392-409. doi:10.1080/09602011.2015.1036890

8. Blampied NM. Reliable Change and the Reliable Change Index in the context of evidence-based practice: A tutorial review. Published online 2016. Accessed January 16, 2023. https://ir.canterbury.ac.nz/handle/10092/13399

9. Guerdoux-Ninot E, Martin S, Jailliard A, Brouillet D, Trouillet R. Validity of the French Prospective and Retrospective Memory Questionnaire (PRMQ) in healthy controls and in patients with no cognitive impairment, mild cognitive impairment and Alzheimer disease. *J Clin Exp Neuropsychol*. 2019;41(9):888-904. doi:10.1080/13803395.2019.1625870

10. Nasreddine ZS, Phillips NA, Bédirian V, et al. The Montreal Cognitive Assessment, MoCA: a brief screening tool for mild cognitive impairment. *J Am Geriatr Soc*. 2005;53(4):695-699.

11. Lezak MD, Howieson DB, Bigler ED, et al. *Neuropsychological Assessment*. Oxford University Press; 2012.

12. Mitrushina M, Boone KB, Razani J, D’Elia LF. *Handbook of Normative Data for Neuropsychological Assessment, 2nd Ed*. Oxford University Press; 2005:xxii, 1029.
