# Supplementary material for: Longitudinal assessment of quality of life, neurocognition, and psychopathology in patients with low-grade glioma on first-line temozolomide: A feasibility study
Source: Neurooncol Adv. 2024 Jun 4;6(1):vdae084. doi: 10.1093/noajnl/vdae084 (PMC11212068; doi:10.1093/noajnl/vdae084)
Supplement: vdae084_suppl_Supplementary_Tables_3 [file vdae084_suppl_Supplementary_Tables_3.docx]

**Supplementary Table 3.** Method to identify a Reliable Change (RC) of the PRMQ score.

| **Formulas to calculate the Reliable Change¹**  *RC* =$\frac{xt1-xt2}{SDIFF}$  *SDIFF* =$\sqrt{2(SEM^{2})}$  *SEM* = $Sd\sqrt{1-Rxx}$  *RC*: Reliable change  *SDIFF*: SD of the errors of measurement of the difference scores  *xt1*: Score at baseline  *xt2*: Score at time X  *SEM*: Standard Error of Measurement  *Sd*: Standard error of the reference dataset  *Rxx*: Test-retest reliability of the measure (Cronbach’s alpha) in the reference data-set  **Conlusion:**  If RC ∈ ]-∞ ; 1.96] U [1.96 ; +∞[, the change should not be due to a measurement error p<0.5 | **PRMQ values of the reference dataset**²  **Retrospective memory (RM):**  *Rxx* = 0.79  *Sd* = 5.5435  *SEM* = 2.54  *SDIFF*= 3.59  **Prospective memory (PM):**  *Rxx* = 0.88  *Sd* = 6.3008  *SEM* = 2.18  *SDIFF*= 3.09 |
| --- | --- |

^1^Blampied, N.M. (2016) Reliable Change and the Reliable Change Index in the context of evidence-based practice: A tutorial review. Wellington, New Zealand: New Zealand Psychological Society Annual Conference 2016, 1-4 Sep 2016.

²Standard errors were estimated from Table 2 of the following article: Guerdoux-Ninot E, Martin S, Jailliard A, Brouillet D, Trouillet R. Validity of the French Prospective and Retrospective Memory Questionnaire (PRMQ) in healthy controls and in patients with no cognitive impairment, mild cognitive impairment and Alzheimer disease. J Clin Exp Neuropsychol. 2019 Nov;41(9):888-904. doi: 10.1080/13803395.2019.1625870. Epub 2019 Aug 6. PMID: 31382847. The Cronbach’s alpha values are from the same article.
