# Supplementary material for: Longitudinal assessment of quality of life, neurocognition, and psychopathology in patients with low-grade glioma on first-line temozolomide: A feasibility study
Source: Neurooncol Adv. 2024 Jun 4;6(1):vdae084. doi: 10.1093/noajnl/vdae084 (PMC11212068; doi:10.1093/noajnl/vdae084)
Supplement: vdae084_suppl_Supplementary_Tables_4 [file vdae084_suppl_Supplementary_Tables_4.docx]

**Supplementary Table 4.** Grade 3 or 4 adverse events of TMZ (CTCAE-NCT criteria).

| **Event** | **Number of patients (%)** |
| --- | --- |
| Any adverse event | 6 (24.0) |
| Hematological event:  Thrombocytopenia  Lymphopenia  Neutropenia | 4 (16.0)  1 (4.0)  1 (4.0) |
| Increased γ‑glutamyltransferase | 1 (4.0) |
| Vomiting | 1 (4.0) |
| Fever | 1 (4.0) |
