# Supplementary figures and images for: Longitudinal assessment of quality of life, neurocognition, and psychopathology in patients with low-grade glioma on first-line temozolomide: A feasibility study
Source: Neurooncol Adv. 2024 Jun 4;6(1):vdae084. doi: 10.1093/noajnl/vdae084 (PMC11212068; doi:10.1093/noajnl/vdae084)

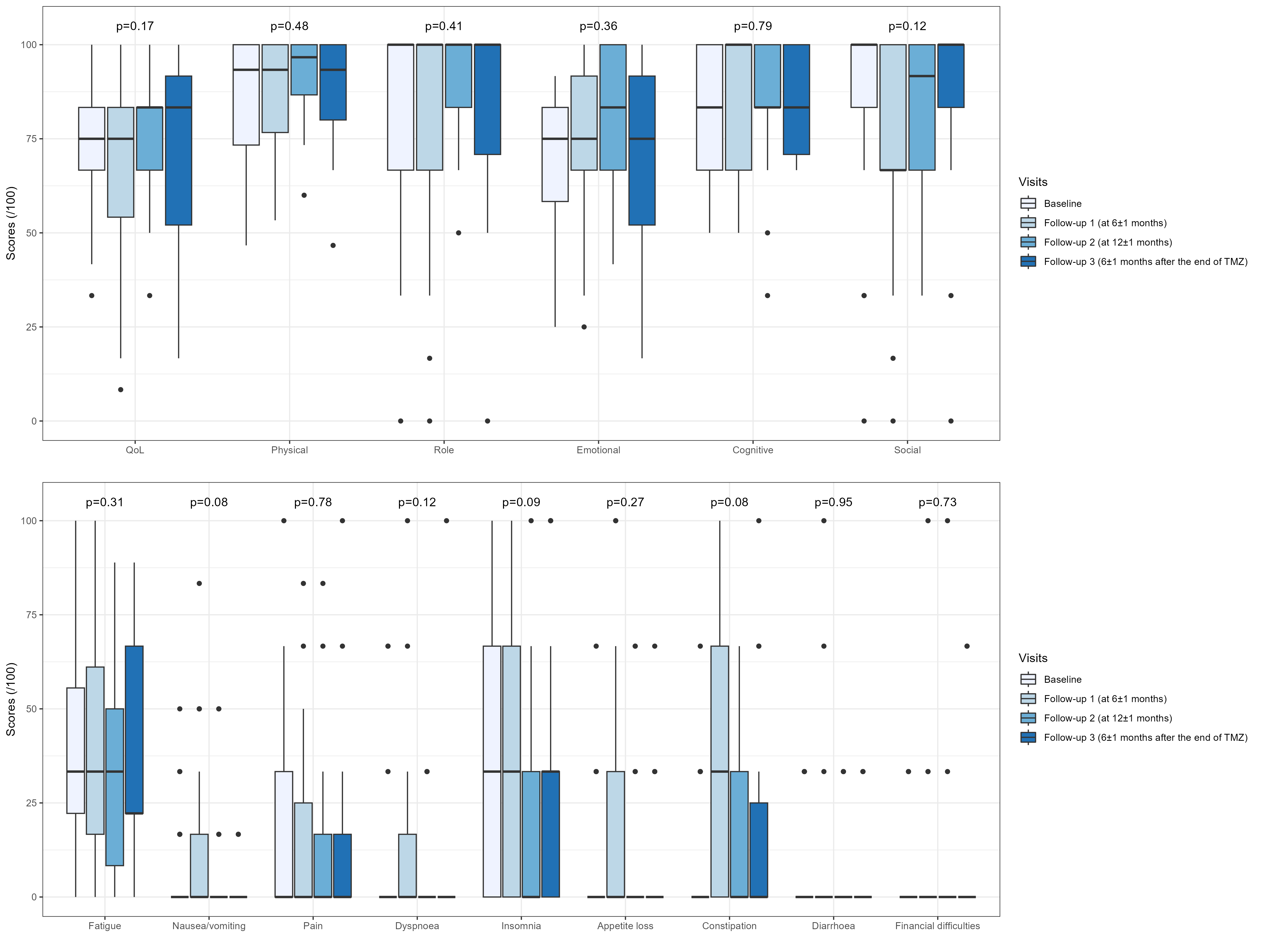

Supplement: vdae084_suppl_Supplementary_Figures_1 [file vdae084_suppl_Supplementary_Figures_1.tiff]

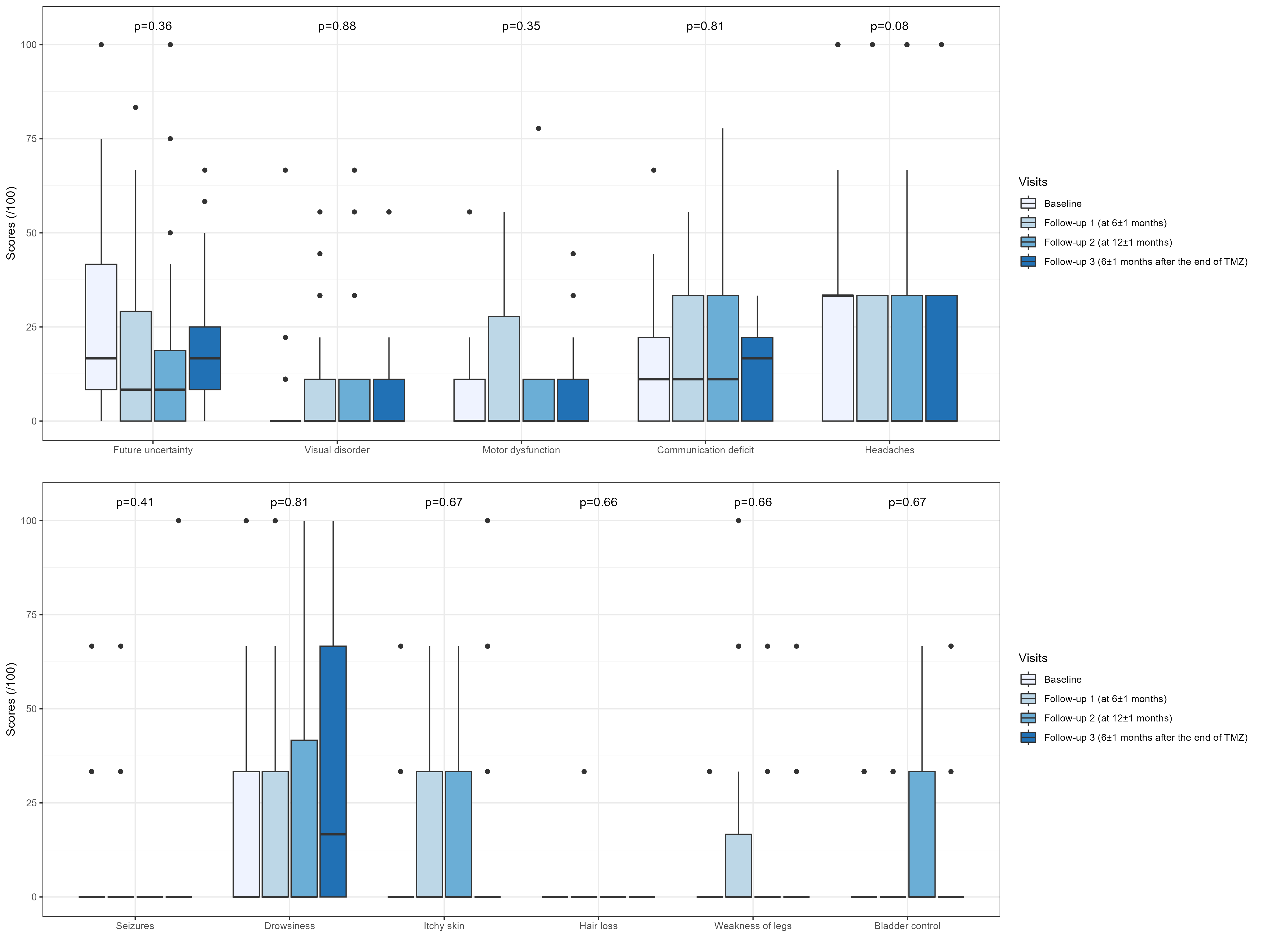

Supplement: vdae084_suppl_Supplementary_Figures_2 [file vdae084_suppl_Supplementary_Figures_2.tiff]

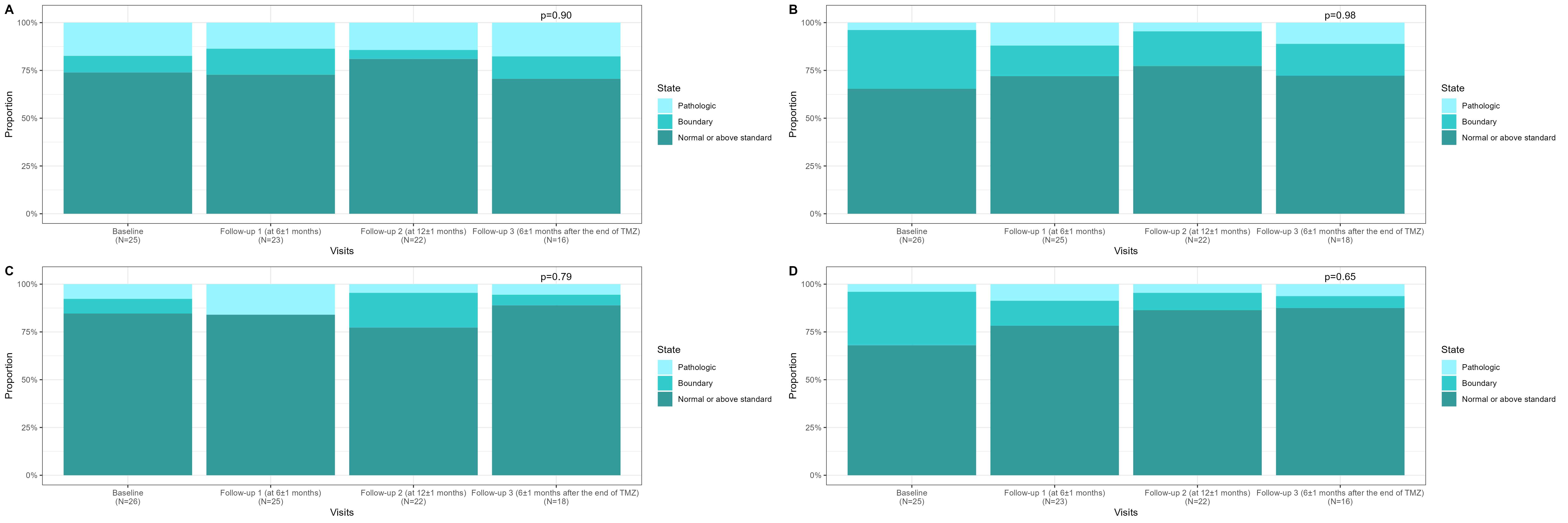

Supplement: vdae084_suppl_Supplementary_Figures_3 [file vdae084_suppl_Supplementary_Figures_3.tiff]

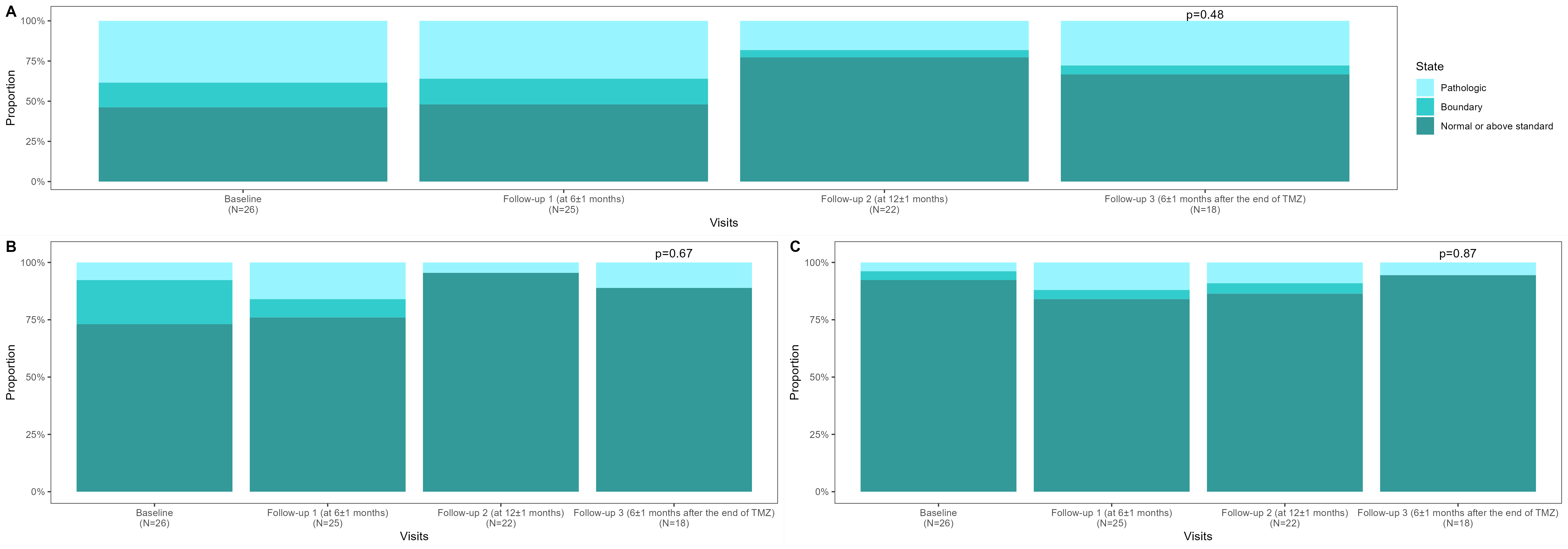

Supplement: vdae084_suppl_Supplementary_Figures_4 [file vdae084_suppl_Supplementary_Figures_4.tiff]
