# Supplementary material for: Longitudinal assessment of quality of life, neurocognition, and psychopathology in patients with low-grade glioma on first-line temozolomide: A feasibility study
Source: Neurooncol Adv. 2024 Jun 4;6(1):vdae084. doi: 10.1093/noajnl/vdae084 (PMC11212068; doi:10.1093/noajnl/vdae084)
Supplement: vdae084_suppl_Supplementary_Tables_5 [file vdae084_suppl_Supplementary_Tables_5.docx]

**Supplementary Table 5.** Changes in quality of life during the follow-up.

|  | **Visit** | | | | | | | | **Test** |  |  |  |  |  |  |  |  |
| --- | --- | --- | --- | --- | --- | --- | --- | --- | --- | --- | --- | --- | --- | --- | --- | --- | --- |
|  | **Baseline** | | **Follow-up 1** | | **Follow-up 2** | | **Follow-up 3** | |  |  |  |  |  |  |  |  |  |
|  | **N=25** | | **N=23** | | **N=20** | | **N=18** |  |  |  |  |  |  |  |  |  |  |
| **QUALITY OF LIFE MEASURES** | | | | | | | | | |  |  |  |  |  |  |  |  |
| **QLQ-C30** |  |  |  |  |  |  |  |  |  |  |  |  |  |  |  |  |  |
| Global health status |  | |  | |  | |  | |  |  |  |  |  |  |  |  |  |
| N / Nb of missing data | 25 / 0 | | 23 / 0 | | 20 / 0 | | 18 / 0 | | *p=0.17* |  |  |  |  |  |  |  |  |
| Median (min; max) | 75 (33; 100) | | 75 (8; 100) | | 83 (33; 100) | | 83 (17; 100) | |  |  |  |  |  |  |  |  |  |
| Physical functioning |  | |  | |  | |  | |  |  |  |  |  |  |  |  |  |
| N / Nb of missing data | 25 / 0 | | 23 / 0 | | 20 / 0 | | 18 / 0 | | *p=0.48* |  |  |  |  |  |  |  |  |
| Median (min; max) | 93 (47; 100) | | 93 (53; 100) | | 97 (60; 100) | | 93 (47; 100) | |  |  |  |  |  |  |  |  |  |
| Role functioning |  | |  | |  | |  | |  |  |  | | |  | | |  |
| N / Nb of missing data | 25 / 0 | | 23 / 0 | | 20 / 0 | | 18 / 0 | | *p=0.41* |  |  |  |  |  |  |  |  |
| Median (min; max) | 100 (0; 100) | | 100 (0; 100) | | 100 (50; 100) | | 100 (0; 100) | |  |  |  |  |  |  |  |  |  |
| Emotional functioning |  | |  | |  | |  | |  |  |  | | |  | | |  |
| N / Nb of missing data | 25 / 0 | | 23 / 0 | | 20 / 0 | | 18 / 0 | | *p=0.36* |  |  |  |  |  |  |  |  |
| Median (min; max) | 75 (25; 92) | | 75 (25; 100) | | 83 (42; 100) | | 75 (17; 100) | |  |  |  |  |  |  |  |  |  |
| Cognitive functioning |  | |  | |  | |  | |  |  |  | | |  | | |  |
| N / Nb of missing data | 25 / 0 | | 23 / 0 | | 20 / 0 | | 18 / 0 | | *p=0.79* |  |  |  |  |  |  |  |  |
| Median (min; max) | 83 (50; 100) | | 100 (50; 100) | | 83 (33; 100) | | 83 (67; 100) | |  |  |  |  |  |  |  |  |  |
| Social functioning |  | |  | |  | |  | |  |  |  | | |  | | |  |
| N / Nb of missing data | 25 / 0 | | 23 / 0 | | 20 / 0 | | 18 / 0 | | *p=0.12* |  |  |  |  |  |  |  |  |
| Median (min; max) | 100 (0; 100) | | 67 (0; 100) | | 92 (33; 100) | | 100 (0; 100) | |  |  |  |  |  |  |  |  |  |
| Fatigue |  | |  | |  | |  | |  |  |  | | |  | | |  |
| N / Nb of missing data | 25 / 0 | | 23 / 0 | | 20 / 0 | | 18 / 0 | | *p=0.31* |  |  |  |  |  |  |  |  |
| Median (min; max) | 33 (0; 100) | | 33 (0; 100) | | 33 (0; 89) | | 22 (0; 89) | |  |  |  |  |  |  |  |  |  |
| Nausea and vomiting |  | |  | |  | |  | |  |  |  | | |  | | |  |
| N / Nb of missing data | 25 / 0 | | 23 / 0 | | 20 / 0 | | 18 / 0 | | *p=0.08* |  |  |  |  |  |  |  |  |
| Median (min; max) | 0 (0; 50) | | 0 (0; 83) | | 0 (0; 50) | | 0 (0; 17) | |  |  |  |  |  |  |  |  |  |
| Pain |  | |  | |  | |  | |  |  |  | | |  | | |  |
| N / Nb of missing data | 25 / 0 | | 23 / 0 | | 20 / 0 | | 18 / 0 | | *p=0.78* |  |  |  |  |  |  |  |  |
| Median (min; max) | 0 (0; 100) | | 0 (0; 83) | | 0 (0; 83) | | 0 (0; 100) | |  |  |  |  |  |  |  |  |  |
| Dyspnea |  | |  | |  | |  | |  |  |  | | |  | | |  |
| N / Nb of missing data | 25 / 0 | | 23 / 0 | | 20 / 0 | | 18 / 0 | | *p=0.12* |  |  |  |  |  |  |  |  |
| Median (min; max) | 0 (0; 67) | | 0 (0; 100) | | 0 (0; 33) | | 0 (0; 100) | |  |  |  |  |  |  |  |  |  |
| Insomnia |  | |  | |  | |  | |  |  |  | | |  | | |  |
| N / Nb of missing data | 25 / 0 | | 23 / 0 | | 20 / 0 | | 18 / 0 | | *p=0.09* |  |  |  |  |  |  |  |  |
| Median (min; max) | 33 (0; 100) | | 33 (0; 100) | | 0 (0; 100) | | 33 (0; 100) | |  |  |  |  |  |  |  |  |  |
| Appetite |  | |  | |  | |  | |  |  |  | | |  | | |  |
| N / Nb of missing data | 25 / 0 | | 23 / 0 | | 20 / 0 | | 18 / 0 | | *p=0.27* |  |  |  |  |  |  |  |  |
| Median (min; max) | 0 (0; 67) | | 0 (0; 100) | | 0 (0; 67) | | 0 (0; 67) | |  |  |  |  |  |  |  |  |  |
| Constipation |  | |  | |  | |  | |  |  |  | | |  | | |  |
| N / Nb of missing data | 25 / 0 | | 23 / 0 | | 20 / 0 | | 18 / 0 | | *p=0.08* |  |  |  |  |  |  |  |  |
| Median (min; max) | 0 (0; 67) | | 33 (0; 100) | | 0 (0; 67) | | 0 (0; 100) | |  |  |  |  |  |  |  |  |  |
| Diarrhea |  | |  | |  | |  | |  |  |  | | |  | | |  |
| N / Nb of missing data | 25 / 0 | | 23 / 0 | | 20 / 0 | | 18 / 0 | | *p=0.95* |  |  |  |  |  |  |  |  |
| Median (min; max) | 0 (0; 33) | | 0 (0; 100) | | 0 (0; 33) | | 0 (0; 33) | |  |  |  |  |  |  |  |  |  |
| Financial difficulties |  | |  | |  | |  | |  |  |  | | |  | | |  |
| N / Nb of missing data | 25 / 0 | | 23 / 0 | | 20 / 0 | | 18 / 0 | | *p=0.73* |  |  |  |  |  |  |  |  |
| Median (min; max) | 0 (0; 33) | | 0 (0; 100) | | 0 (0; 100) | | 0 (0; 67) | |  |  |  |  |  |  |  |  |  |
| **QLQ-BN20** |  | |  | |  | |  | |  |  |  |  |  |  |  |  |  |
| Future uncertainty |  | |  | |  | |  | |  |  | | |  | | |  | |
| N / Nb of missing data | 25 / 0 | | 23 / 0 | | 20 / 0 | | 18 / 0 | | *p=0.36* |  |  |  |  |  |  |  |  |
| Median (min; max) | 17 (0; 100) | | 8 (0; 83) | | 8 (0; 100) | | 17 (0; 67) | |  |  |  |  |  |  |  |  |  |
| Visual disorder |  | |  | |  | |  | |  |  | |  | | |  | | |
| N / Nb of missing data | 25 / 0 | | 23 / 0 | | 20 / 0 | | 18 / 0 | | *p=0.88* |  |  |  |  |  |  |  |  |
| Median (min; max) | 0 (0; 67) | | 0 (0; 56) | | 0 (0; 67) | | 0 (0; 56) | |  |  |  |  |  |  |  |  |  |
| Motor dysfunction |  | |  | |  | |  | |  |  | |  | | |  | | |
| N / Nb of missing data | 25 / 0 | | 23 / 0 | | 20 / 0 | | 18 / 0 | | *p=0.35* |  |  |  |  |  |  |  |  |
| Median (min; max) | 0 (0; 56) | | 0 (0; 56) | | 0 (0; 78) | | 0 (0; 44) | |  |  |  |  |  |  |  |  |  |
| Communication deficit |  | |  | |  | |  | |  |  | |  | | |  | | |
| N / Nb of missing data | 25 / 0 | | 23 / 0 | | 20 / 0 | | 18 / 0 | | *p=0.81* |  |  |  |  |  |  |  |  |
| Median (min; max) | 11 (0; 67) | | 11 (0; 56) | | 11 (0; 78) | | 17 (0; 33) | |  |  |  |  |  |  |  |  |  |
| Headache |  | |  | |  | |  | |  |  | |  | | |  | | |
| N / Nb of missing data | 25 / 0 | | 23 / 0 | | 20 / 0 | | 18 / 0 | | *p=0.08* |  |  |  |  |  |  |  |  |
| Median (min; max) | 33 (0; 100) | | 0 (0; 100) | | 0 (0; 100) | | 0 (0; 100) | |  |  |  |  |  |  |  |  |  |
| Seizures |  | |  | |  | |  | |  |  | |  | | |  | | |
| N / Nb of missing data | 25 / 0 | | 23 / 0 | | 20 / 0 | | 18 / 0 | | *p=0.41* |  |  |  |  |  |  |  |  |
| Median (min; max) | 0 (0; 67) | | 0 (0; 67) | | 0 (0; 0) | | 0 (0; 100) | |  |  |  |  |  |  |  |  |  |
| Drowsiness |  | |  | |  | |  | |  |  | |  | | |  | | |
| N / Nb of missing data | 25 / 0 | | 23 / 0 | | 20 / 0 | | 18 / 0 | | *p=0.81* |  |  |  |  |  |  |  |  |
| Median (min; max) | 0 (0; 100) | | 0 (0; 100) | | 0 (0; 100) | | 17 (0; 100) | |  |  |  |  |  |  |  |  |  |
| Hair loss |  | |  | |  | |  | |  |  | |  | | |  | | |
| N / Nb of missing data | 25 / 0 | | 23 / 0 | | 20 / 0 | | 18 / 0 | | *p=0.67* |  |  |  |  |  |  |  |  |
| Median (min; max) | 0 (0; 0) | | 0 (0; 33) | | 0 (0; 0) | | 0 (0; 0) | |  |  |  |  |  |  |  |  |  |
| Itchy skin |  | |  | |  | |  | |  |  | |  | | |  | | |
| N / Nb of missing data | 25 / 0 | | 23 / 0 | | 20 / 0 | | 18 / 0 | | *p=0.66* |  |  |  |  |  |  |  |  |
| Median (min; max) | 0 (0; 67) | | 0 (0; 67) | | 0 (0; 67) | | 0 (0; 100) | |  |  |  |  |  |  |  |  |  |
| Weakness of legs |  | |  | |  | |  | |  |  | |  | | |  | | |
| N / Nb of missing data | 25 / 0 | | 23 / 0 | | 20 / 0 | | 18 / 0 | | *p=0.66* |  |  |  |  |  |  |  |  |
| Median (min; max) | 0 (0; 33) | | 0 (0; 100) | | 0 (0; 67) | | 0 (0; 67) | |  |  |  |  |  |  |  |  |  |
| Bladder control |  | |  | |  | |  | |  |  | |  | | |  | | |
| N / Nb of missing data | 25 / 0 | | 23 / 0 | | 20 / 0 | | 18 / 0 | | *p=0.67* |  |  |  |  |  |  |  |  |
| Median (min; max) | 0 (0; 33) | | 0 (0; 33) | | 0 (0; 67) | | 0 (0; 67) | |  |  |  |  |  |  |  |  |  |
|  |  | **Visit** | | | | | |  | **Test** |  |  |  |  |  |  |  |  |
|  | **Baseline** | | **Follow-up 1** | | **Follow-up 2** |  | **Follow-up 3** | |  |  |  |  |  |  |  |  |  |
|  | **N=26** | | **N=26** | | **N=23** | | **N=21** | |  |  |  |  |  |  |  |  |  |
| **MFI-20** |  |  |  |  |  |  |  |  |  |  |  |  |  |  |  |  |  |
| General fatigue |  |  |  |  |  |  |  |  |  |  |  |  |  |  |  |  |  |
| N / Nb of missing data | 26 / 0 | | 24 / 2 | | 22 / 1 | | 19 / 2 | | *p*=0.78 |  |  |  |  |  |  |  |  |
| Median (min; max) | 30 (11; 61) | | 29 (9; 59) | | 27 (9; 54) | | 27 (10; 61) | |  |  |  |  |  |  |  |  |  |
| Mental fatigue |  |  |  |  |  |  |  |  |  |  |  |  |  |  |  |  |  |
| N / Nb of missing data | 26 / 0 | | 24 / 2 | | 22 / 1 | | 19 / 2 | | *p*=0.45 |  |  |  |  |  |  |  |  |
| Median (min; max) | 16 (6; 34) | | 16 (6; 32) | | 13 (6; 30) | | 20 (6; 35) | |  |  |  |  |  |  |  |  |  |
| Reduced activities |  |  |  |  |  |  |  |  |  |  |  |  |  |  |  |  |  |
| N / Nb of missing data | 26 / 0 | | 24 / 2 | | 22 / 1 | | 19 / 2 | | *p*=0.46 |  |  |  |  |  |  |  |  |
| Median (min; max) | 7 (3; 20) | | 8 (3; 19) | | 7 (3; 21) | | 6 (3; 21) | |  |  |  |  |  |  |  |  |  |
| Motivation |  |  |  |  |  |  |  |  |  |  |  |  |  |  |  |  |  |
| N / Nb of missing data | 26 / 0 | | 24 / 2 | | 22 / 1 | | 19 / 2 | | *p*=0.67 |  |  |  |  |  |  |  |  |
| Median (min; max) | 3 (2; 12) | | 5 (2; 13) | | 4 (2; 12) | | 3 (2; 10) | |  |  |  |  |  |  |  |  |  |
| Total score |  |  |  |  |  |  |  |  |  |  |  |  |  |  |  |  |  |
| N / Nb of missing data | 26 / 0 | | 24 / 2 | | 22 / 1 | | 19 / 2 | | *p*=0.82 |  |  |  |  |  |  |  |  |
| Median (min; max) | 60 (24; 111) | | 58 (20; 120) | | 59 (20; 110) | | 61 (21; 112) | |  |  |  |  |  |  |  |  |  |
| **FACT-COG** |  | |  | |  | |  | |  |  |  |  |  |  |  |  |  |
| Perceived cognitive impairment |  | |  | |  | |  | |  |  |  |  |  |  |  |  |  |
| N / Nb of missing data | 26 / 0 | | 24 / 2 | | 22 / 1 | | 19 / 2 | | *p*=0.87 |  |  |  |  |  |  |  |  |
| Median (min; max) | 56 (30; 71) | | 59 (26; 71) | | 56 (19; 72) | | 59 (33; 69) | |  |  |  |  |  |  |  |  |  |
| Comments from others |  | |  | |  | |  | |  |  |  |  |  |  |  |  |  |
| N / Nb of missing data | 26 / 0 | | 24 / 2 | | 22 / 1 | | 18 / 3 | | *p*=0.67 |  |  |  |  |  |  |  |  |
| Median (min; max) | 15 (9; 16) | | 16 (11; 16) | | 16 (4; 16) | | 16 (12; 16) | |  |  |  |  |  |  |  |  |  |
| Perceived cognitive abilities |  | |  | |  | |  | |  |  |  |  |  |  |  |  |  |
| N / Nb of missing data | 26 / 0 | | 23 / 3 | | 22 / 1 | | 19 / 2 | | *p*=0.88 |  |  |  |  |  |  |  |  |
| Median (min; max) | 21 (9; 26) | | 21 (10; 28) | | 21 (4; 26) | | 20 (8; 28) | |  |  |  |  |  |  |  |  |  |
| Impact on quality of life |  | |  | |  | |  | |  |  |  |  |  |  |  |  |  |
| N / Nb of missing data | 26 / 0 | | 24 / 2 | | 22 / 1 | | 19 / 2 | | *p*=0.48 |  |  |  |  |  |  |  |  |
| Median (min; max) | 12 (0; 16) | | 14 (0; 16) | | 15 (2; 16) | | 12 (6; 16) | |  |  |  |  |  |  |  |  |  |
| **PRMQ** |  | |  | |  | |  | |  |  |  |  |  |  |  |  |  |
| Prospective memory |  | |  | |  | |  | |  |  |  |  |  |  |  |  |  |
| N / Nb of missing data | 20 / 6 | | 23 / 2 | | 20 / 2 | | 19 / 1 | | *p*=0.90 |  |  |  |  |  |  |  |  |
| Median (min; max) | 16 (11; 34) | | 17 (11; 29) | | 17 (10; 30) | | 18 (13; 29) | |  |  |  |  |  |  |  |  |  |
| Retrospective memory |  | |  | |  | |  | |  |  |  |  |  |  |  |  |  |
| N / Nb of missing data | 20 / 6 | | 23 / 2 | | 20 / 2 | | 19 / 1 | | *p*=0.48 |  |  |  |  |  |  |  |  |
| Median (min; max) | 16 (9; 37) | | 15 (9; 23) | | 16 (9; 34) | | 16 (10; 30) | |  |  |  |  |  |  |  |  |  |

The p-values were adjusted using the Benjamini and Hochberg method.
