# Supplementary material for: Longitudinal assessment of quality of life, neurocognition, and psychopathology in patients with low-grade glioma on first-line temozolomide: A feasibility study
Source: Neurooncol Adv. 2024 Jun 4;6(1):vdae084. doi: 10.1093/noajnl/vdae084 (PMC11212068; doi:10.1093/noajnl/vdae084)
Supplement: vdae084_suppl_Supplementary_Tables_6 [file vdae084_suppl_Supplementary_Tables_6.docx]

**Supplementary Table 6.** Changes in Z-scores of the neurocognitive measures during the follow-up.

|  | **Visit** | | | | | | | | **Test** |
| --- | --- | --- | --- | --- | --- | --- | --- | --- | --- |
|  | **Baseline** | | **Follow-up 1** | | **Follow-up 2** | | **Follow-up 3** | |  |
|  | **N=26** | | **N=25** | | **N=22** | | **N=20** | |  |
| **Working memory** |  |  |  |  |  |  |  |  | *p = 0.35* |
| N / Nb of missing data | 25 / 1 | | 22 / 3 | | 22 / 0 | | 19 / 1 | |  |
| Median (min; max) | -0.39 (-2.33; 0.84) | | -0.84 (-2.33; 1.63) | | -0.54 (-2.33; 1.04) | | -0.68 (-1.65; 1.28) | |  |
| **Processing speed** |  |  |  |  |  |  |  |  | *p = 0.07* |
| N / Nb of missing data | 23 / 3 | | 22 / 3 | | 20 / 2 | | 18 / 2 | |  |
| Median (min; max) | -0.25 (-2.33; 1.65) | | -0.32 (-2.33; 1.04) | | -0.25 (-2.33; 1.28) | | 0.00 (-2.33; 1.28) | |  |
| **Language** |  | |  | |  | |  | | *p = 0.66* |
| N / Nb of missing data | 25 / 1 | | 21 / 4 | | 21 / 1 | | 17 / 3 | |  |
| Median (min; max) | -0.87 (-2.26; 1.31) | | -0.68 (-2.09; 1.05) | | -0.84 (-2.26; 0.66) | | -0.49 (-2.09; 0.31) | |  |
| **Episodic memory** |  |  |  |  |  |  |  |  | *p = 0.07* |
| N / Nb of missing data | 26 / 0 | | 25 / 0 | | 22 / 0 | | 18 / 2 | |  |
| Median (min; max) | -1.37 (-6.29; 1.37) | | -0.60 (-3.28; 1.79) | | -0.71 (-4.42; 1.70) | | 0.24 (-4.14; 1.57) | |  |
| **Attention** |  |  |  |  |  |  |  |  | *p = 0.35* |
| N / Nb of missing data | 26 / 0 | | 23 / 2 | | 20 / 2 | | 19 / 1 | |  |
| Median (min; max) | -0.44 (-6.62; 0.93) | | -0.18 (-7.49; 1.15) | | -0.20 (-2.47; 1.06) | | -0.18 (-8.17; 0.47) | |  |
| **Executive functioning** |  |  |  |  |  |  |  |  | *p = 0.35* |
| N / Nb of missing data | 26 / 0 | | 25 / 0 | | 22 / 0 | | 20 / 0 | |  |
| Median (min; max) | -0.45 (-1.84; 1.02) | | -0.04 (-2.01; 0.93) | | -0.21 (-1.38; 1.08) | | -0.07 (-4.15; 1.01) | |  |

*Notes.* The p-values were adjusted using the Benjamini and Hochberg method.
