# Supplementary material for: Longitudinal assessment of quality of life, neurocognition, and psychopathology in patients with low-grade glioma on first-line temozolomide: A feasibility study
Source: Neurooncol Adv. 2024 Jun 4;6(1):vdae084. doi: 10.1093/noajnl/vdae084 (PMC11212068; doi:10.1093/noajnl/vdae084)
Supplement: vdae084_suppl_Supplementary_Tables_7 [file vdae084_suppl_Supplementary_Tables_7.docx]

**Supplementary Table 7.** Changes of psychological scales scores during the follow-up.

|  | **Visit** | | | | | **Test*** |
| --- | --- | --- | --- | --- | --- | --- |
|  | **Baseline** | **Follow-up 1** | **Follow-up 2** | **Follow-up 3** | |  |
|  | **N=26** | **N=26** | **N=23** | **N=21** |  |  |
| **BDI-II** |  |  |  |  | |  |
| N / N of missing data | 26 / 0 | 25 / 1 | 22 / 1 | 18 / 3 | | *p=0.46* |
| Median (min; max) | 11 (1; 26) | 10 (0; 25) | 6 (0; 35) | 5 (0; 35) | |  |
| **STAI-Y: State Anxiety** |  |  |  |  | |  |
| N / N of missing data | 26 / 0 | 25 / 1 | 22 / 1 | 18 / 3 | | *p=0.17* |
| Median (min; max) | 32 (20; 46) | 31 (20; 70) | 24 (20; 70) | 27 (20; 42) | |  |
| **STAXI-II: State Anger** |  |  |  |  | |  |
| N / N of missing data | 26 / 0 | 25 / 1 | 22 / 1 | 18 / 3 | | *p=0.79* |
| Median (min; max) | 16 (15; 33) | 17 (15; 36) | 15 (15; 34) | 15 (15; 38) | |  |

*The p-values were adjusted using the Benjamini and Hochberg method.
